# Purkinje Cell Loss and Changes in Basket Cell Morphology in Essential Tremor and Other Cerebellar Degenerative Diseases: A Postmortem Study of 332 Brains

**DOI:** 10.1007/s12311-026-02075-2

**Published:** 2026-08-25

**Authors:** Ethan Wainman, Tomer O. Guy, Nora Hernandez, Phyllis L. Faust, Elan D. Louis

**Affiliations:** 1 Department of Neurology, University of Texas Southwestern Medical Center, 5323 Harry Hines Blvd, Dallas, TX 75390- 8813 USA; 2 Department of Pathology and Cell Biology, Columbia University Irving Medical Center and New York Presbyterian Hospital, New York, NY USA; 3 Peter O’Donnell Jr. Brain Institute, University of Texas Southwestern Medical Center, Dallas, TX USA

**Keywords:** Essential tremor, Cerebellum, Purkinje cells, Basket cells, Spinocerebellar ataxia, Pathology

## Abstract

Essential tremor (ET) is among the most prevalent neurological diseases. Postmortem studies have demonstrated Purkinje cell (PC) loss in ET. In addition, PCs and their micro-circuitry in the cerebellar cortex—including basket cells and climbing fibers—display several characteristic pathological features in these studies. While several studies in ET have focused on the interactions between PCs and climbing fibers, less has been written about basket cells and their interactions with PCs (i.e., formation of empty baskets and hypertrophy of basket cell axonal processes around the PC soma). These changes are not unique to ET, yet scarcely anything has been written about basket cells and their interactions with PCs more broadly across disorders of cerebellar degeneration. We examined the relationship between three metrics (PC linear density, percentage of empty baskets, basket plexus rating [a measure of basket cell axonal process hypertrophy]) in 332 brains: 178 ET, 56 with a range of spinocerebellar ataxias, 13 Friedreich’s ataxia, and 85 controls. We hypothesized that there would be overlapping but not uniform findings across these disorders. In all groups, PC linear density significantly negatively correlated with percentage of empty baskets, although to differing degrees. Basket plexus ratings were significantly associated with PC linear density and percentage of empty baskets only in some diseases. In summary, PC linear density, percentage of empty baskets and basket plexus rating were associated metrics across a range of cerebellar degenerative conditions and even in control brains. However, their relationships with one another were variable.

**Supplementary Information:**

The online version contains supplementary material available at https://doi.org/10.1007/s12311-026-02075-2.

## Introduction

Essential tremor (ET) is among the most often-encountered neurological diseases [1, 2], and the most common form of cerebellar degeneration [3, 4]. Despite its extraordinarily high prevalence, our understanding of its underlying pathophysiology remains limited.

A number of studies have demonstrated loss of Purkinje cells (PCs) in ET [5–7]. In addition, PCs and their micro-circuitry in the cerebellar cortex—including basket cells and climbing fibers—display several characteristic pathological features in ET postmortem studies [6–8]. These and other changes can be used to reliably distinguish ET brains from control brains [9], underscoring that PCs and these neighboring and interacting neuronal populations undergo a series of interconnected physiological and morphological changes in the setting of this chronic, progressive disease [6–8]. 

While several studies have focused on the interactions between PCs and climbing fibers in ET [10–12], less has been written about basket cells and their interactions with PCs in ET. Basket cells are interneurons that release gamma-aminobutyric acid (GABA), and their cell bodies are located in the molecular layer of the cerebellar cortex. Their axonal collaterals have an intimate connection with PCs, forming a perisomal basket plexus around the PC soma and a pinceau structure around the PC axon initial segment [13]. The structure of these perisomal basket plexuses provides a window into the health of the neighboring PC population with which they interact. First, in the setting of PC loss, these baskets may appear as “empty baskets” (i.e., basket plexuses lacking a PC soma) [14]. Second, in the setting of PC degeneration, the perisomal baskets may undergo a reactive process where basket cell axonal processes are likely recruited from neighboring PCs that are degenerating and/or lost, forming a densely tangled basket plexus around remaining PC soma [15]. 

The above-mentioned changes in the PC and basket cell populations are not specific to ET and have been observed to various degrees in other disorders of cerebellar degeneration [6, 7]. However, disorders of cerebellar degeneration are not homogeneous; these disorders vary considerably in terms of the extent of PC loss and degree of empty basket formation, and they likely differ in terms of the pace and extent of reactive changes observed in the basket cell population [6, 7]. For example, ET is a slowly progressive disease, often extending over decades [16, 17], whereas several spinocerebellar ataxias (SCA) progress more rapidly. Furthermore, the breadth and extent of PC degeneration is expressed quite differently across these disorders.

As noted above, surprisingly little has been written about basket cells and their interactions with PCs in ET or more broadly across disorders of cerebellar degeneration. The morphology of these basket plexuses provides a window into the health of the neighboring PC population with which they interact as well as broader clues about the nature of the underlying pathological cascade that unfolds across different disorders of cerebellar degeneration. Leveraging data from a large brain banking effort of ET and a diverse range of disorders of cerebellar degeneration, in this study, we aimed to further elucidate the relationship between PC loss and basket cell morphology across a range of cerebellar degenerative disorders, including ET, forms of SCA that relatively spare the cerebellar cortex (SCA3) and those that do not spare the cerebellar cortex (e.g., SCA 1, 2, and 6), and Friedreich’s ataxia (FA). Our sample also included normal controls, among whom there is some observed PC loss with age [5]. Our overarching goal was to understand more fully how PC pathology and basket cell reactive processes converge or diverge across a broad range of cerebellar degenerative diseases. Our hypothesis was that there would be overlapping but not uniform findings across this panoply of disorders.

## Methods

### Study Subjects

In all, 332 brains were involved in this analysis. All participants provided written informed consent. The Columbia University and University of Texas Southwestern Medical Center Institutional Review Boards approved the study protocol, approval number STU-2020-0564.

The ET brains (*n* = 178) were obtained from the Essential Tremor Centralized Brain Repository (ETCBR) (New York, NY), a joint brain bank program between investigators from University of Texas Southwestern Medical Center and Columbia University.

We were able to obtain all available SCA brains (*n* = 16 SCA1, 9 SCA2, 16 SCA3, 7 SCA6, 5 SCA7, 2 SCA8, and 1 SCA14, total *n* = 56) and FA brains (*n* = 13) from multiple brain repositories: 40 from a hereditary ataxia specimen repository at the Veterans Affairs Medical Center in Albany, NY; 9 from the National Institutes of Health NeuroBioBank at the University of Maryland, Baltimore, MD; 12 from the Center for NeuroGenetics Ataxia Brain Bank at the University of Florida, Gainesville, FL; 6 from the University of Washington, Seattle, WA; 1 from the Sheffield Biorepository at the University of Sheffield, Sheffield, UK; and 1 from the New York Brain Bank, New York, NY.

Brains from control participants (*n* = 85) were also obtained from various sources to achieve a 2:1 ratio with ET cases. We selected [1] older controls whose age most closely approximated those of our ET cases as well as [2] a smaller subset of younger controls to provide some age-matching with several of the other diseases in which death occurs at younger ages. Sixty-eight were obtained from the New York Brain Bank, from individuals who had been prospectively followed at the Alzheimer’s Disease Research Center or the Washington Heights Inwood Columbia Aging Project, Columbia University. These individuals were followed with longitudinal clinical examinations, and were clinically free of neurodegenerative disorders, including ET, Alzheimer’s disease, Parkinson’s disease (PD), or progressive supranuclear palsy. Eleven controls were obtained from the University of Miami, Miami, FL, and 6 were obtained from the University of Maryland, Baltimore, MD.

In comparison to our 2023 study of cerebellar cortex histopathology in ET and other neurodegenerative motor disorders [7], this study assembled 78% more ET cases (178 compared to 100), 70% more controls (85 compared to 50), more SCA3 (16 compared to 13), and more other SCAs (40 compared to 34).

### Tissue Processing and Evaluation

For the ET cases collected by our group, after a participant’s death, their brain was procured and delivered to the ETCBR, where it underwent a full neuropathological evaluation. First, a brain weight (grams) and postmortem interval (time, in hours, elapsed between death and placement of the brain into a cold room or on ice) were obtained. Next, seventeen standardized tissue blocks were dissected from each formalin-fixed brain, and 7 μm thick paraffin embedded sections were stained with Luxol fast blue/hematoxylin and eosin (LH&E) [6]. Control brains obtained through the New York Brain Bank were processed in the same manner. Cerebellar tissues received from other brain repositories were from the same standard parasagittal region as harvested at the New York Brain Bank; paraffin embedded tissue sections or a fixed cerebellar tissue block that was subsequently embedded in paraffin were provided.

### PC Linear Density

For quantifying PC loss in the 332 brains, a parasagittal cerebellar tissue block (3 × 20 × 25 mm) was obtained from a region ~ 1.5 cm lateral to the midline, which included the anterior and posterior quadrangulate lobules and dentate nucleus. Using a 7 μm-thick LH&E stained paraffin section from this block, PC bodies were counted in at least fifteen non-overlapping 100x magnification microscopic fields across the tissue section. The average number of PC bodies/field was divided by the microscopic field diameter (mm/field) to obtain PCs/mm.

### Empty Baskets

To quantify empty baskets, which provided an indirect measure of PC loss [14], a dual immunohistochemical stain for calbindinD_28k_ (to label PCs bodies) and glutamic acid decarboxylase (GAD) (to label basket cell processes) was performed on another 7 μm-thick paraffin section from the same cerebellar block. Within each of 4 randomly selected, non-contiguous regions across the cerebellar paraffin section, at least 200 total “full” (containing a PC soma) or “empty baskets” (no PC soma) were counted at 200–400x magnification to calculate the percentage of empty baskets. See Fig. 1 in Lee et al. [14]. for example of empty baskets.

**Fig. 1:**
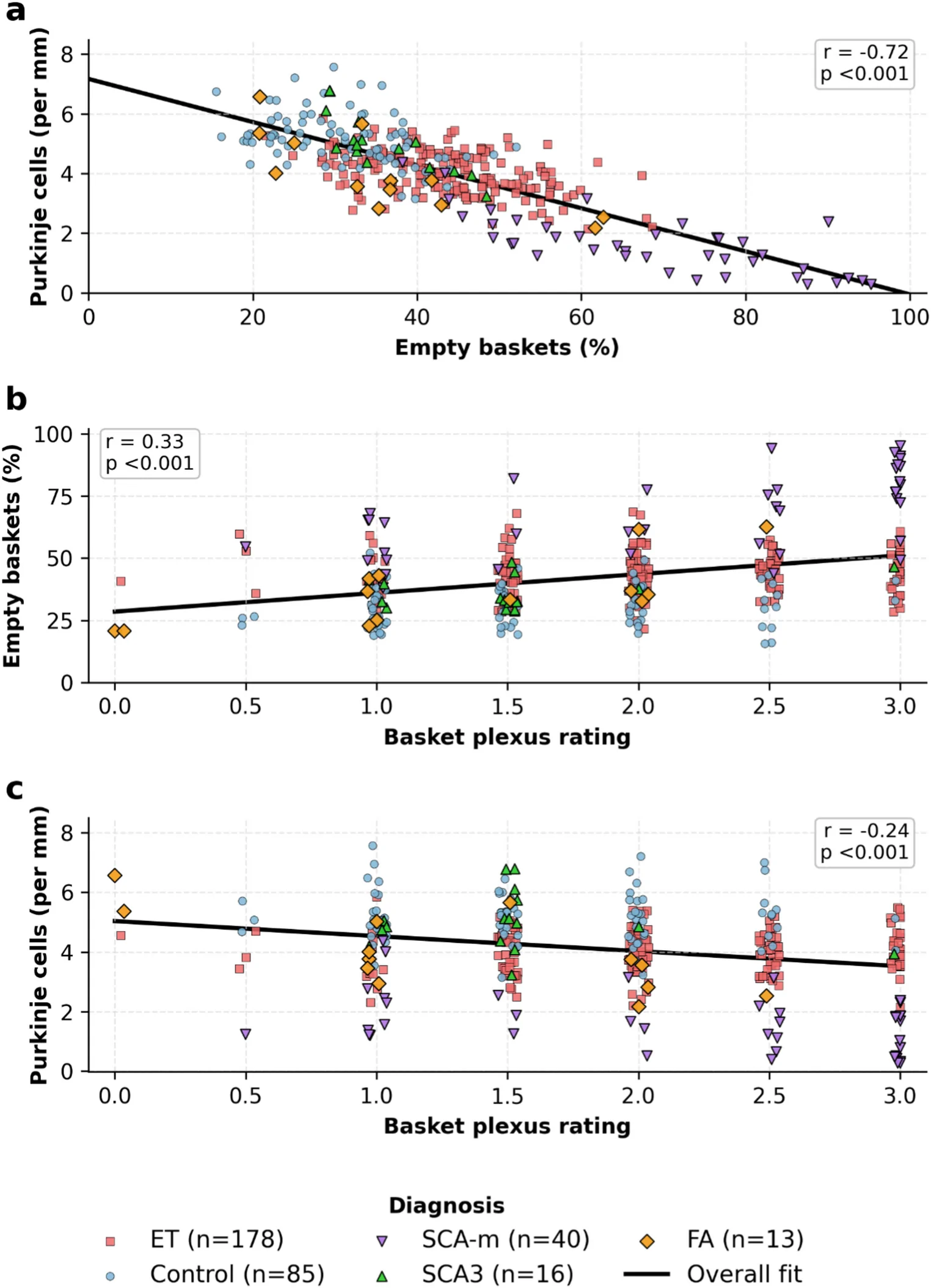
Correlations (with all diagnosis groups combined) for (**a**) PC linear density (mm^− 1^) and empty baskets, (**b**) empty baskets and basket plexus ratings, and (**c**) PC linear density (mm^− 1^) and basket plexus ratings

### Basket Plexus Rating

Another 7 μm-thick paraffin section of cerebellum was stained by a modified Bielschowksy silver method, which highlights basket cell processes around PC somas. A semi-quantitative scale was employed to reflect the density of basket cell axonal plexus around PC bodies. The scale ranges from 0 to 3 with 0.5-increment ratings, where 0 represents few or no discernible processes, 1 a sparse number of processes, 2 a moderate number of processes, and 3 a dense tangle of processes, and intermediate values representing a midpoint between the corresponding descriptions [15]. See Fig. 1 in Erickson-Davis [15] for examples of semi-quantitative ratings of the basket plexus.

### Statistical Analyses

Statistical analysis was performed in SPSS (Version 29; IBM Corp, 2023). Basic demographic features are reported for each diagnosis with their proportions, means, and standard deviations. Each of the diagnostic groups was statistically compared against control participants. Categorical variables were compared using Chi-squares. For continuous variables, we tested for normality using Kolmogorov-Smirnov test. If normally distributed, the variables were compared using Student’s *t* test. If not normally distributed, we compared groups using the Mann-Whitney test (non-parametric). For ordinal variables, we compared groups using the Mann-Whitney test. Bivariate correlation analyses were performed first, and then partial correlation coefficients were performed in which we adjusted for age.

In line with prior research, for our primary analyses, we divided SCA cases for analyses into two groups: [1] SCA1, SCA2, SCA6, SCA7, SCA8, and SCA14 in one group, which are all characterized by marked PC loss; and [2] SCA3 in its own group, because PCs are largely spared [7]. We termed the former group “SCA-multiple” (SCA-m). In supplemental analyses, we separated each SCA subtype (i.e., SCA1 SCA2, SCA6, SCA7, SCA8, and SCA14). Means and standard deviations of the three cerebellar pathological features of focus were calculated for each of the groups. Each of the three metrics (PC linear density, percentage of empty baskets, basket plexus rating) was graphed against one another for all diagnoses combined as well by each diagnosis group, and Spearman’s correlations with 95% confidence intervals (CIs) were calculated.

## Results

### Demographic Characteristics Across Diagnoses

The study involved 332 participants with different forms of cerebellar degeneration as well as controls − 178 ET, 16 SCA3, 13 FA, 40 SCA-m (16 SCA1, 9 SCA2, 7 SCA6, 5 SCA7, 2 SCA8, and 1 SCA14) and 85 controls. Each group differed from controls with respect to age (Table 1). This was not unexpected - as noted above, we selected older controls whose age most closely approximated those of our ET cases as well as a smaller subset of younger controls to provide some age-matching with several of the other diseases in which death occurs at younger ages. Data on each of the six SCA subtypes are also presented, demonstrating a heterogeneous range of demographic features (i.e., age and sex) (Supplemental Table 1).

**Table 1:** Participant demographic and pathological features

	Control	FA	SCA3	ET	SCA-m
Sample size, *n*	85	13	16	178	40
Age of death, years	78.7 ± 14.4 [82.0]	41.4 ± 18.1 [37.0]	56.0 ± 10.4 [53.0]	89.5 ± 6.0 [90.0]	60.6 ± 16.2 [53.0]
		***p*** **< 0.001**	***p*** **< 0.001**	***p*** **< 0.001**	***p*** **< 0.001**
Female Sex (%)	39 (45.9)	8 (61.5)	6 (37.5)	115 (64.6)	14 (35.0)
		*p* = 0.29	*p* = 0.54	***p*** **= 0.004**	*p* = 0.25
Purkinje cell linear density, mm^− 1^	5.2 ± 0.9 [5.1]	4.0 ± 1.3 [3.8]	5.0 ± 1.0 [4.9]	4.0 ± 0.7 [4.1]	1.6 ± 1.0 [1.6]
		***p*** **= 0.002**	*p* = 0.40	***p*** **< 0.001**	***p*** **< 0.001**
Empty Baskets (%)	31.1 ± 8.0 [31.2]	36.4 ± 13.6 [35.3]	35.9 ± 6.4 [33.0]	43.7 ± 8.8 [43.9]	68.0 ± 16.1 [68.5]
		*p* = 0.28	***p*** **= 0.04**	***p*** **< 0.001**	***p*** **< 0.001**
Basket plexus rating	1.6 ± 0.6 [1.5]	1.3 ± 0.8 [1.0]	1.5 ± 0.5 [1.5]	2.0 ± 0.6 [2.0]	2.2 ± 0.8 [2.5]
		*p* = 0.20	*p* = 0.36	***p*** **< 0.001**	***p*** **< 0.001**

All values are presented as mean ± SD [median] unless otherwise specified. Statistical comparisons are with controls

Bolded values are statistically significant (*p* < 0.05)

Categorical variables were compared using Chi-squares. For continuous variables, we tested for normality using Kolmogorov-Smirnov test. If normally distributed, the variables were compared using Student’s *t-*test. If not normally distributed, we compared groups using the Mann-Whitney test (non-parametric). For ordinal variables, we compared groups using the Mann-Whitney test

*ET* Essential Tremor, *FA* Friedreich’s Ataxia, *SCA3* Spinocerebellar Ataxia Type 3, *SCA-m* Spinocerebellar Ataxia multiple

### PC Linear Density, Empty Baskets and Basket Plexus Ratings Across Diagnoses

In comparison with controls, PC linear densities were lower in FA (*p* = 0.002), ET (*p* < 0.001), and SCA-m (*p* < 0.001); participants with SCA3 did not differ from controls with respect to PC counts (Table 1).

When compared to controls, the following diagnosis groups had higher percentages of empty baskets: SCA3 (*p* = 0.04), ET (*p* < 0.001), and SCA-m (*p* < 0.001); participants with FA did not differ to a significant degree from controls with respect to percentage of empty baskets (Table 1).

The following diagnosis groups had higher basket plexus ratings than controls: ET (*p* < 0.001) and SCA-m (*p* < 0.001) (Table 1). Participants with SCA3 did not differ from controls with respect to basket plexus rating and participants with FA had lower basket plexus ratings than controls.

Data for each of the six SCA subtypes are also presented, indicating heterogeneity of findings with respect to PC linear density, percentage of empty baskets and basket plexus rating, although small n’s in some groups made formal comparisons difficult (Supplemental Table 1). Notwithstanding, PC loss and empty basket formation seemed to be less marked in SCA1.

### Correlations of Cerebellar Pathological Metrics (Across All Diagnostic Groups)

We first examined associations between PC linear density, empty baskets and basket plexus rating metrics across all diagnostic groups combined to identify overall data trends independent of clinical diagnosis. When all diagnosis groups were combined, all three of the metrics were correlated with one another: PC linear density was inversely correlated with percentage of empty baskets (*r* = -0.72, *p* < 0.001), basket plexus ratings were positively correlated with percentage of empty baskets (*r* = 0.33, *p* < 0.001), and basket plexus ratings were negatively correlated with PC linear density (*r* = -0.24, *p* < 0.001) (Fig. 1). Adjusting for age, these correlations were highly similar: PC linear density was inversely correlated with percentage of empty baskets (*r* = -0.79, *p* < 0.001), basket plexus ratings were positively correlated with percentage of empty baskets (*r* = 0.37, *p* < 0.001), and basket plexus ratings were negatively correlated with PC linear density (*r* = -0.27, *p* < 0.001).

### Correlations of Cerebellar Pathological Metrics (Stratified by Diagnostic Group)

**Fig. 2:**
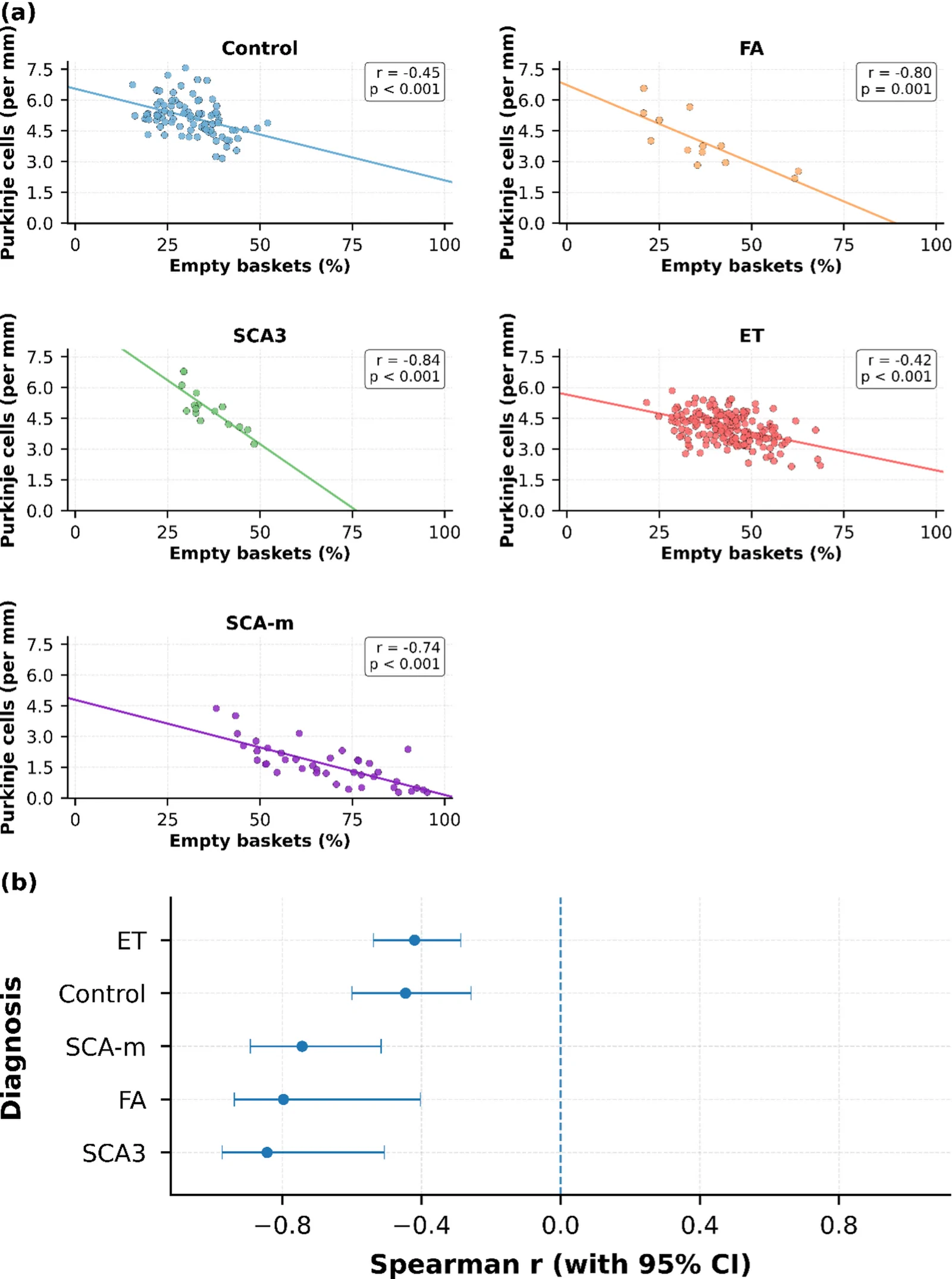
Correlations between Purkinje cell linear density and empty baskets in each diagnosis group. (**a**) In each diagnosis group, PC linear density (mm^− 1^) and empty baskets were inversely correlated. The inverse correlation was significant in all groups. (**b**) Spearman r values (represented by the plotted point) with 95% CIs (represented by the horizontal error bars) are shown for each diagnosis

**Fig. 3:**
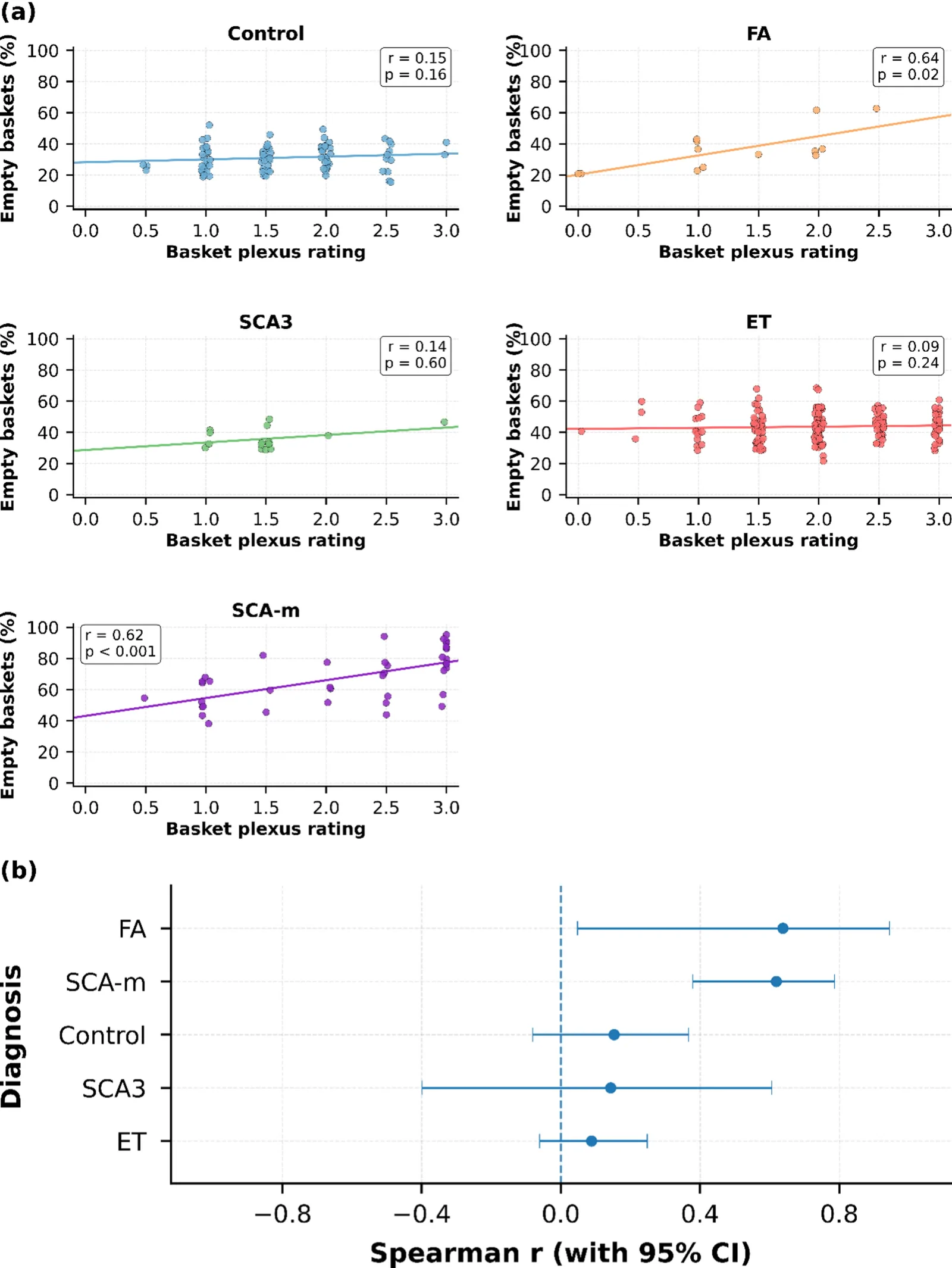
Correlations between basket plexus ratings and percentage of empty baskets in each diagnosis group. (**a**) In all groups, basket ratings were positively correlated with percentage of empty baskets to some degree. The correlation was significant in two groups: FA and SCA-m. There was no significant correlation between these two metrics for controls, SCA3, and ET. (**b**) Spearman r values (represented by the plotted point) with 95% CIs (represented by the horizontal error bars) are shown for each diagnosis

**Fig. 4:**
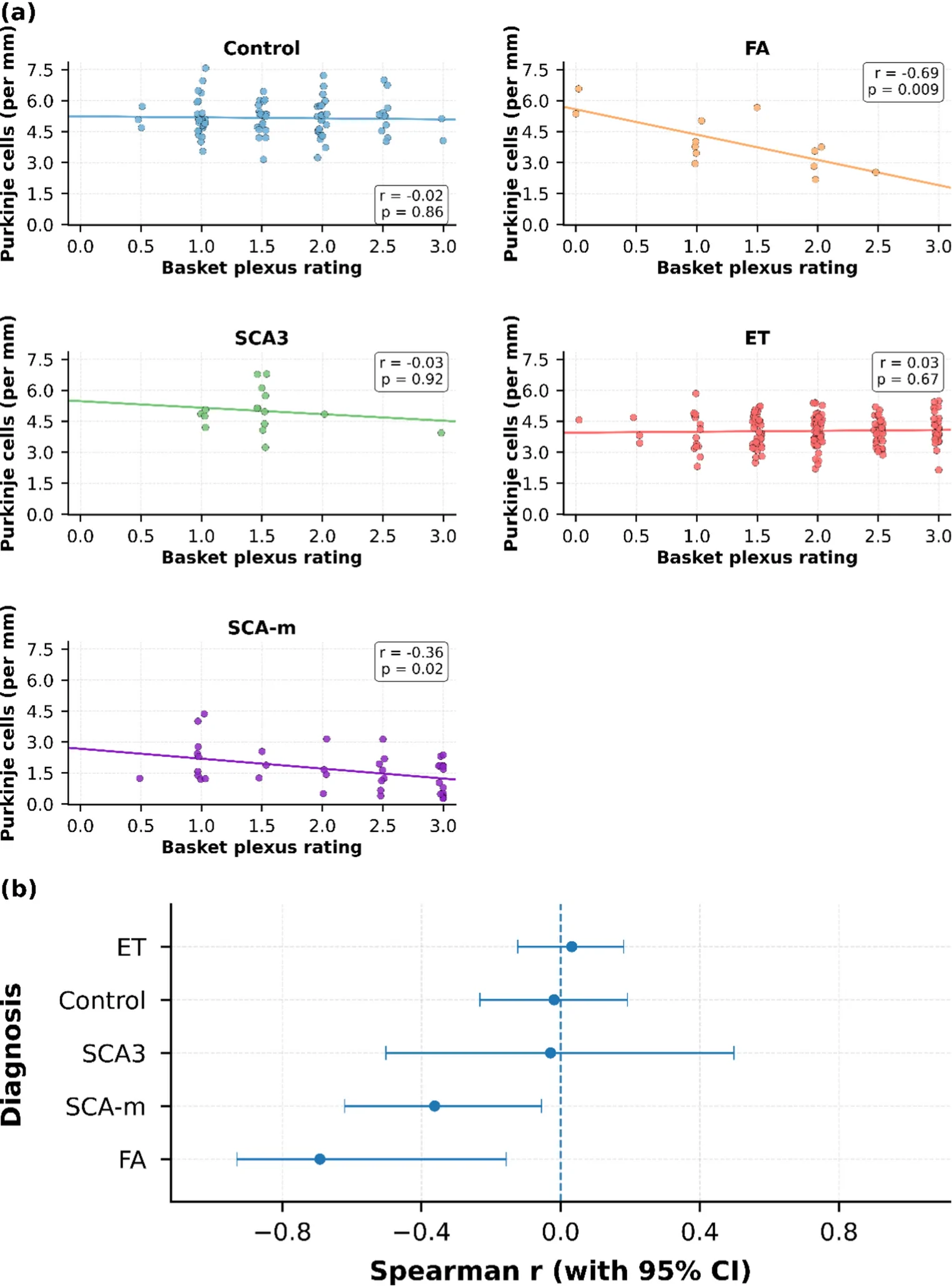
Correlations between Purkinje cell linear density and basket plexus ratings in each diagnosis group. (**a**) In all groups except for ET, basket ratings were negatively correlated with PC linear density (mm^− 1^) to some degree, although not always to a significant degree. The correlation was significant in two groups: SCA-m and FA. In ET, basket ratings and PC linear density (mm^− 1^) are slightly positively correlated. There was no significant correlation between these two metrics for ET, controls, and SCA3. (**b**) Spearman r values (represented by the plotted point) with 95% CIs (represented by the horizontal error bars) are shown for each diagnosis

We next performed these same correlations between metrics within each diagnostic group to observe disease specific patterns (Figs. 2, 3 and 4; Table 2).

**Table 2:**
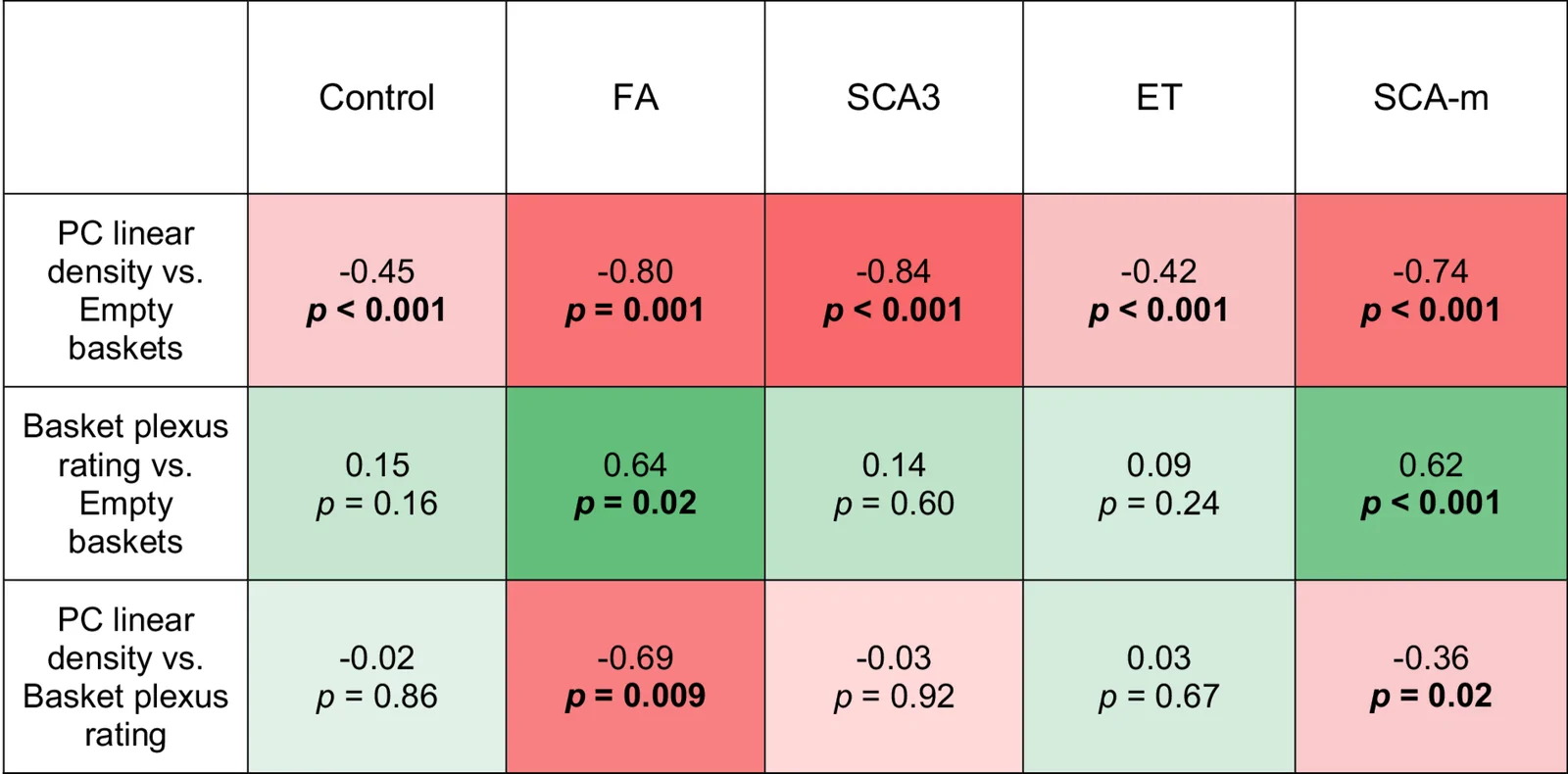
Spearman correlations between PC linear density, empty baskets, and basket plexus ratings in all diagnosis groups

Values shown are Spearman r correlation values, with corresponding p values underneath

Bolded values are statistically significant (*p* < 0.05)

Color grading is based on the strength of the correlation, where deeper shade of red = more negative (closer to -1) and deeper shade of green = more positive (closer to +1)

*ET* Essential Tremor, *FA* Friedreich’s Ataxia, *SCA3* Spinocerebellar Ataxia Type 3, *SCA-m* Spinocerebellar Ataxia multiple

PC linear density was significantly inversely correlated with percentage of empty baskets in all diagnostic groups, although to different degrees. The correlation was highest in SCA3, FA, and SCA-m and lower in control and ET, although 95% CIs overlapped for most groups (Fig. 2).

Basket plexus ratings were positively correlated with percentage of empty baskets in all diagnosis groups, but only to a significant degree in FA and SCA-m. The 95% CIs overlapped for most groups (Fig. 3).

Basket plexus ratings were inversely correlated with PC linear density in all groups except for ET, in which they were slightly positively correlated. The correlation was only significant in FA and SCA-m. The 95% CIs overlapped for most groups (Fig. 4).

There were significant differences in age among our diagnostic groups, and age is a well-established factor associated with PC loss and age-related cerebellar degeneration. Partial correlation coefficients that adjusted for age yielded similar results, with all significant associations observed in the unadjusted analyses observed here as well (Supplemental Table 2), supporting that these are disease specific patterns and not driven solely by age.

We further stratified SCA-m into its component diagnoses (SCA1, 2, 3, 7, 8 and 14) and present correlation data in Supplemental Table 3. The small n in some groups lessened the statistical power of these analyses; however, PC linear density was broadly inversely associated with percentage of empty baskets across most subtypes.

## Discussion

Beyond the pathological findings, a plethora of clinical [18–20], physiological [21], and imaging [22] studies support a cerebello-thalamo-cortical model of circuit dysfunction in ET. Postmortem data robustly implicate changes in PCs and surrounding neuronal populations, including basket cells [7]. Aside from ET, the SCAs and other forms of cerebellar degeneration also display varying degrees of cerebellar degeneration on postmortem examination [6]. We know, then, that the pathological features investigated in this study—reduced PC linear density, empty baskets, and basket plexus ratings—are not necessarily a distinct feature of ET. Leveraging a large body of postmortem data across a diverse range of disorders of cerebellar degeneration, as well as a large group of control brains for contextualization, we aimed to further elucidate the relationship between PC loss and basket cell morphologies. Our overarching goal was to understand more fully how PC pathology and basket cell reactive processes converge or diverge across a broad range of cerebellar degenerative diseases.

For these analyses, we focused on three primary metrics: [1] PC linear density, as reduced PC linear density relative to controls is a repeated finding in many studies of ET [2, 9, 23], percentage of empty baskets, as the remnants of basket cell synaptic contacts onto once-present PCs may provide an indirect marker of PC loss [14] and [3], basket plexus ratings, a semi-quantitative measure of the hypertrophy of basket cell axonal plexuses, which is a process hypothesized to occur when basket cell processes converge onto particular PCs, following the death or damage of neighboring PCs—providing another potential secondary marker of PC injury and death [15]. 

Across all clinical diagnostic groups, we observed statistically significant differences in PC linear density, empty baskets, and basket plexus ratings. Furthermore, there was a consistently significant inverse relationship between PC linear density and empty baskets within each condition (Table 2). The magnitude of this correlation for ET cases was similar to that of controls (Fig. 2b), while FA, SCA3 and SCA-m had correlations of higher magnitude. Correlations of basket plexus rating with either PC linear density or empty baskets were not significant in either ET or controls (Figs. 3b and 4b), even though ET cases differ significantly from controls in all three of these metrics (Table 1). That basket plexus rating is elevated in ET compared to controls but not associated with other markers of PC loss/damage in ET may indicate that basket cell axonal hypertrophy is not driven solely by the extent of PC loss, as will be discussed in greater detail below. Only in SCA-m and FA were basket plexus ratings significantly associated with both percentage of empty baskets and PC linear density (Figs. 3b and 4b; Table 2). Small within-group sample sizes, as demonstrated by the width of several of the CIs (Figs. 3b and 4b), and simple sampling issues (e.g., natural variation) could also have accounted for some of our null results.

In our primary analyses, we combined the majority of SCA subtypes; however, in additional analyses, we presented separable data by each subtype. These groups presented a heterogeneous picture, with differences in PC linear density and basket cell pathology, as might be expected given the known genetic, clinical and pathological heterogeneity across these diseases. In general, the correlation between PC linear density and percentage empty baskets was most robust across groups, as was observed when the groups were combined. The small n (< 10) in all but one of these groups should be noted; hence, any comparisons between groups should be approached with caution.

Understanding the degree to which primary markers of PC degeneration are associated with other markers of degeneration or the remodeling of neighboring neuronal populations has multiple clinical and mechanistic implications. Knowledge of and estimates of the magnitude of the associations add to a growing compendium of disease-specific pathological findings [7] which is useful for differentiating and diagnosing conditions with similar cerebellar involvement at post-mortem. Further, the degree to which these metrics are coupled or decoupled may provide clues to underlying mechanistic processes. For example, our results raise multiple intriguing possibilities: the relatively modest association between PC linear density and percentage of empty baskets in ET compared to other diseases such as SCAs and FA may be suggestive of heterogenous mechanisms driving basket cell remodeling. The genetic and environmental factors that underlie ET are not well established; however, they are not identical to those that underlie these other disorders. It stands to reason, then, that the unfolding downstream cascade of abnormal molecular events in ET will not be identical to that of these disorders. The precise mechanism whereby this cascade eventually results in PC loss in ET is not known. Similarly, it is not known whether the underlying genetic and environmental factors in ET produce similarly unique changes in the biology of the neuronal populations that neighbor PCs in ET. The temporal component of ET may be important as well —since ET cases live longer on average, it may be that basket cells have considerable time to undergo a more dynamic remodeling processes following PC insult. Alternatively, a more rapid rate of PC degeneration, as seen in SCA, might result in more extensive remodeling [24]. What is clear is that the pattern of basket plexus pathology is not uniform across these diseases, and these differences raise important questions about the underlying processes. What processes drive basket plexus hypertrophy and what is its time course? Are empty baskets a permanent feature of a degenerated cerebellar cortex, or do they undergo remodeling and eventual dissolution over time?

We present a model whereby basket plexus hypertrophy may arise from recruitment and remodeling of basket cell axonal processes following degeneration or loss of neighboring PCs. However, as noted above, some of our data do not support this model; e.g., in this sample of ET cases, and in SCA3, basket plexus ratings were not significantly correlated with either PC linear density or empty basket percentage. These findings raise the possibility that basket cell remodeling may be regulated by mechanisms beyond simple PC loss. Alternative biological mechanisms for formation of empty baskets could include alterations in GABAergic neurotransmission, neuroinflammatory processes, dysregulation of the SEMA3A/NRP1 signaling pathway and/or activity-dependent structural plasticity within cerebellar microcircuits.

Much is known about the molecular basis of basket plexus formation from a developmental perspective. Studies of mouse mutants with targeted deletions of the axon guidance molecule Semaphorin 3 A (SEMA3A, secreted by PCs) [25] or its receptor Neuropilin-1(NRP-1) [26] demonstrate that PC–derived SEMA3A initially attracts and orients NRP-1–expressing basket cell axons towards their targets. NRP-1 then mediates subcellular target recognition at the PC axon initial segment by trans-synaptic interaction with neurofascin-186, a L1 immunoglobulin family cell adhesion molecule that is required for the formation of the pinceau and is expressed by both PCs and basket cells. Notably, inactivation of neurofascin-186 in adult PCs destabilizes the axon initial segment and pinceau structure, potentially implicating this molecule in neurodegenerative processes within the cerebellum [27–29]. Basket cell pinceau size also varies with cerebellar zonal organization, being larger around PCs with higher firing rates (e.g., aldolase C-negative PCs); however, this size differential depended on PC neurotransmission rather than basket cell GABAergic input onto PCs, pointing to a target cell–driven mechanism of structural plasticity [30]. Nonetheless, early hyperactivity of molecular layer interneurons potentiates PC degeneration in SCA1 mice [31], indicating a complex interplay between PC and interneuron activity in both normal physiology and disease progression. Whether these developmental and organizational processes persist across the lifespan — and whether their disruption may relate to the pathological findings observed in this study — remains an important question for future investigation [25, 30]. 

Some of the metrics we study in detail here have been examined in prior work, including assessments of their associations. In an earlier study of 32 ET cases and 21 controls, there was a marginal inverse association between PC loss and basket plexus rating (*p* = 0.06) [15], and in another study of 127 brains (controls, ET, SCA3, SCA-m), PC linear density, % empty baskets and basket plexus rating were all correlated with one another, although the analyses did not stratify by diagnosis as we have done here [14]. 

We recognize certain limitations to this study. First, although the number of brains was large, compared to ET and controls, there were relatively fewer cases of SCA-m, SCA3, and FA. This may have made it more difficult to test associations in our correlation analyses; however, despite this, robust correlations were detected for SCA-m and FA (Figs. 2B, 3B and 4B) and even for SCA3 (Fig. 2B). Second, the basket cell rating is a semi-quantitative ordinal scale; thus, compared to the other two continuous measures utilized in the study, presents certain limitations. Ordinal measures provide limited resolution compared to continuous measures, given the lack of equivalence between unit measurements. In addition, our studies have demonstrated that the basket plexus rating varied considerably even within a single slide, meaning that the assigned rating is an overall rating of a heterogenous and variable process [32]. This creates statistical noise. Third, while a prior study in ET demonstrated an increased number of pinceau processes that abnormally targeted PC axon segments more distal to the axon initial segment [33], we have not examined this morphologic change in the large cohort of brains in this study. Fourth, we restricted our analysis to a particular region of the hemispheric cerebellar cortex but encourage further exploration of other regions. Fifth, we did not exclude cases based on extra-cerebellar pathologies such as Lewy pathology or Alzheimer’s type changes. Previously, it has been shown that there is not an association between these metrics of PC loss in ET cases and the presence and/or severity of any accompanying Alzheimer’s type neuropathological changes or Lewy body pathology [5, 32]. The study had numerous strengths including: [1] a large number (> 300) of brains [2], inclusion of multiple cerebellar degenerative diseases [3], inclusion of controls of varied ages, and [4] the fact that the research question has not been studied to any great degree in ET, in controls or in the setting of other cerebellar degenerative diseases.

## Summary

In summary, this paper supports that PC linear density, empty baskets and basket plexus rating were associated metrics across a range of cerebellar degenerative conditions and even in the normal control brains. However, their relationships with one another were variable, and seem to have been influenced by the underlying diagnosis. These differing inter-relationships provide interesting questions for future study.

## Supplementary Information

Below is the link to the electronic supplementary material.

Supplementary File 1 (DOCX 21.6 KB)

## Data Availability

Data are available by contacting the corresponding authors.
