# Supplementary material for: Purkinje Cell Loss and Changes in Basket Cell Morphology in Essential Tremor and Other Cerebellar Degenerative Diseases: A Postmortem Study of 332 Brains
Source: Cerebellum. 2026 Aug 25;25(5):133. doi: 10.1007/s12311-026-02075-2 (PMC13506590; doi:10.1007/s12311-026-02075-2)
Supplement: Supplementary file 1 [file 12311_2026_2075_MOESM1_ESM.docx]

Supplemental Table 1: Demographic and Pathological Features of Participants with SCA1, 2, 6, 7, 8 and 14

|  | SCA1 | SCA2 | SCA6 | SCA7 | SCA8 | SCA14 |
| --- | --- | --- | --- | --- | --- | --- |
| Sample size, n | 16 | 9 | 7 | 5 | 2 | 1 |
| Age of death, years | 58.6 ± 12.8 [60.0] | 51.1 ± 14.1 [48.0] | 78.3 ± 5.9 [78.0] | 54.2 ± 19.9 [49.0] | 60.0 * [60.0] | 89 [89.0] |
|  |  |  |  |  |  |  |
| Female Sex (%) | 4 (25.0) | 3 (33.3) | 4 (57.1) | 3 (60.0) | 0 (0.0.0) | 0 (0.0) |
|  |  |  |  |  |  |  |
| Purkinje cell linear density, mm^-1^ | 2.2 ± 1.0 [1.9] | 1.2 ± 0.9 [1.1] | 1.2 ± 0.9 [1.0] | 1.9 ± 0.7 [1.7] | 0.9 ± 0.5 [0.9] | 0.4 [0.4] |
|  |  |  |  |  |  |  |
| Empty Baskets (%) | 59.5 ± 12.4 [58.3] | 75.7 ± 17.2 [77.5] | 82.5 ± 10.3 [87.0] | 55.9 ± 8.5 [51.8] | 78.9 ± 19.2 [78.9] | 74.0 [74.0] |
|  |  |  |  |  |  |  |
| Basket plexus rating | 2.0 ± 0.9 [2.3] | 2.6 ± 0.5 [2.5] | 2.6 ± 0.8 [3.0] | 1.4 ± 0.6 [1.0] | 2.0 ± 1.4 [2.0] | 3.0 [3.0] |
|  |  |  |  |  |  |  |
| All values are presented as mean ± SD [median] unless otherwise specified.  *Data missing on one case.  SCA = Spinocerebellar Ataxia | | | | | |  |

Supplemental Table 2: Partial correlations between PC linear density, empty baskets, and basket plexus ratings in all diagnosis groups

|  | Control | FA | SCA3 | ET | SCA-m |
| --- | --- | --- | --- | --- | --- |
| PC linear density vs. Empty baskets | -0.28 | -0.91 | -0.66 | -0.46 | -0.76 |
|  | **p = 0.01** | **p = 0.001** | **p = 0.007** | **p < 0.001** | **p < 0.001** |
| Basket plexus rating vs. Empty baskets | 0.19 | 0.72 | 0.22 | 0.06 | 0.58 |
|  | p = 0.09 | **p = 0.008** | p = 0.435 | p = 0.45 | **p < 0.001** |
| PC linear density vs. Basket plexus rating | -0.12 | -0.76 | 0.05 | 0.05 | -0.41 |
|  | p = 0.29 | **p = 0.004** | p = 0.85 | p = 0.52 | **p = 0.01** |

Supplemental Table 3: Spearman correlations between PC linear density, empty baskets, and basket plexus ratings in participants with SCA1, 2, 6, 7, 8, and 14

|  | SCA1 | SCA2 | SCA6 | SCA7 | SCA8 | SCA14 |
| --- | --- | --- | --- | --- | --- | --- |
|  | n = 16 | n = 9 | n = 7 | n = 5 | n = 2 | n = 1 |
| PC linear density vs. Empty baskets | -0.62 | -0.92 | -0.36 | -1.00 | NA | NA |
|  | **p = 0.01** | **p < 0.001** | p = 0.43 | **p = 0.01** |  |  |
| Basket plexus rating vs. Empty baskets | 0.55 | -0.34 | 0.09 | -0.29 | NA | NA |
|  | **p = 0.03** | p = 0.37 | p = 0.85 | p = 0.64 |  |  |
| PC linear density vs. Basket plexus rating | -0.29 | 0.38 | 0.13 | 0.29 | NA | NA |
|  | p = 0.28 | p = 0.31 | p = 0.78 | p = 0.64 |  |  |
